# Chromosome positioning from activity-based segregation

**DOI:** 10.1093/nar/gkt1417

**Published:** 2014-01-22

**Authors:** Nirmalendu Ganai, Surajit Sengupta, Gautam I. Menon

**Affiliations:** ^1^Department of Physics, Nabadwip Vidyasagar College, Nabadwip, Nadia 741302, India, ^2^TIFR Centre for Interdisciplinary Sciences, 21 Brundavan Colony, Narsingi, Hyderabad 500075, India, ^3^Centre for Advanced Materials, Indian Association for the Cultivation of Science, Jadavpur, Kolkata 700032, India, ^4^The Institute of Mathematical Sciences, C.I.T. Campus, Taramani, Chennai 600 113, India, ^5^Mechanobiology Institute, National University of Singapore, T-Lab, #10-01, 5A Engineering Drive 1, Singapore 117411, Singapore and ^6^Department of Biological Sciences, National University of Singapore, 14 Science Drive 4, Singapore 117543, Singapore

## Abstract

Chromosomes within eukaryotic cell nuclei at interphase are not positioned at random, since gene-rich chromosomes are predominantly found towards the interior of the cell nucleus across a number of cell types. The physical mechanisms that could drive and maintain the spatial segregation of chromosomes based on gene density are unknown. Here, we identify a mechanism for such segregation, showing that the territorial organization of chromosomes, another central feature of nuclear organization, emerges naturally from our model. Our computer simulations indicate that gene density-dependent radial segregation of chromosomes arises as a robust consequence of differences in non-equilibrium activity across chromosomes. Arguing that such differences originate in the inhomogeneous distribution of ATP-dependent chromatin remodeling and transcription machinery on each chromosome, we show that a variety of non-random positional distributions emerge through the interplay of such activity, nuclear shape and specific interactions of chromosomes with the nuclear envelope. Results from our model are in reasonable agreement with experimental data and we make a number of predictions that can be tested in experiments.

## INTRODUCTION

Studies of the compartmentalization of the cell nucleus trace their origins to the pioneering work of Rabl and Boveri, who first proposed that individual chromosomes within the interphase nucleus of higher eukaryotes were organized into distinct territories (1–3). Recent studies find that chromosomes are not located randomly within the nucleus, quantifying such higher order organization by combining the fluorescent labeling of individual chromosomes with light optical serial sectioning via laser confocal microscopy (4). As an example of such non-random positioning, the gene-rich chromosome 19 is consistently seen toward the interior of the nucleus in human lymphocytes, with the similarly sized but gene-poor chromosome 18 located more peripherally (5). Early measurements of the locations of human chromosomes in nuclei of diploid lymphoblasts inferred a gene density-based radial organization, finding that gene-dense chromosomes were preferentially associated to the nuclear interior (5,6). More broadly, late replicating regions of the genome, containing predominantly non-genic heterochromatin, generally manifest toward the nuclear boundary, whereas gene-rich early replicating euchromatin regions are located closer to the center of the nucleus across multiple cell types (7–10). Finally, studies of nuclear organization in rodents (11), cattle (12) and birds (13) argue for gene density-based radial chromosome positioning, an arrangement which is also conserved across the several million years of evolution separating humans from old-world monkeys (14,15).

The conventional radial arrangement of interior gene-rich euchromatin surrounded by peripheral heterochromatin is strikingly inverted in nuclei of rod cells from the retina of the mouse, a nocturnal mammal, with gene-rich regions now located at the nuclear periphery (16). Alternative forms of radial organization based on chromosome size have been proposed for some cell types, together with a link to nuclear shape, as flatter nuclei appear to favor size-dependent chromosome localization (17). Chromosome–chromosome interactions mediated via the clustering of co-regulated genes at transcription complexes enriched in RNA polymerases, nucleoside triphosphates (NTPs) and transcription factors (transcription factories), arguably favor patterns of relative positioning over absolute positioning (18–21). Nuclear subcompartments as well as the nuclear envelope (NE), composed of the outer and inner nuclear membrane, nuclear pore complexes and the nuclear lamina, a thin filamentous layer of lamin proteins proximate to the inner nuclear membrane, can dynamically organize chromatin (22–26), thus indirectly favoring some patterns of large-scale positioning over others (27,28).

Computational models addressing the positioning problem have so far been unable to convincingly reproduce gene density-dependent radial segregation, let alone more complex patterns of positioning, indicating that they lack a crucial element. Here, we suggest that this missing element is inhomogeneous non-equilibrium activity, a biophysical effect unjustifiably neglected in all models so far. We show that accounting for activity provides a natural solution to two outstanding problems in nuclear architecture: the emergence of a territorial organization of chromosomes and the origins of non-random positional distributions of chromosomes based on gene density. The predictions of the model we describe here are compared with experimental data for distribution functions of human chromosomes 12, 18, 19 and 20 in relatively spherical human lymphocyte nuclei, although we generate model predictions for all chromosomes. These predictions are shown to agree reasonably with the experiments. We discuss how such distributions can be further influenced by specific interactions with the nuclear envelope, exhibiting one mechanism for stabilizing ‘inverted’ radial patterns of chromatin organization.

Chromatin in living cells is, at the physical level, ‘active matter’ i.e. ‘matter driven out of thermal equilibrium through the transduction of energy derived from an internal energy depot or ambient medium, into work performed on the environment’ (29). Inhomogeneous, stochastic forces acting on chromatin, balanced by equal and opposite forces directed toward the surrounding nucleoplasm, are a direct consequence of local energy consuming (ATP-dependent and thus non-equilibrium) enzymatic activity linked to local chromatin remodeling and transcription (30,31). Similar stochastic forces—Brownian forces—are also a feature of the thermal equilibrium state, but the statistical properties of these forces are then ‘homogeneous’, with a magnitude tied to the thermodynamic temperature. Experiments on active matter systems *in vitro*—see e.g. (32–35)—and *in vivo* (36) as well as a substantial body of theoretical work [reviewed in (37–39)], show that active matter can differ in striking ways from matter in thermal equilibrium.

**Figure 1.**
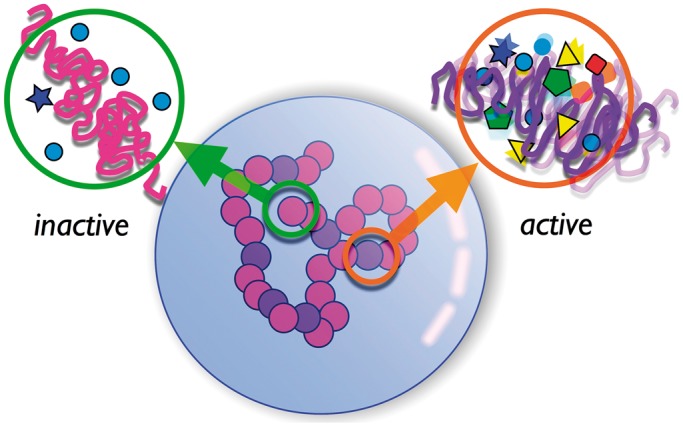
Schematic of model illustrating individual monomers, each providing a coarse-grained description of a 1 Mb section along the chromosome, subdivided into active and inactive, depending on their gene density. The schematic expands out two monomers, one active and one inactive, illustrating how chromatin remodeling and transcription-coupled enzymatic activity translate into differing levels of stochastic forces acting on each monomer. In the expanded figures, colored polygons and spheres represent chromatin remodeling enzymes exerting such stochastic forces on chromatin through their activity. The strength of these forces is captured in our model through an effective temperature—a higher effective temperature means a larger activity.

We make the straightforward assumption, common to a large number of models for active systems, that fluctuations arising from activity can be modeled via a local non-equilibrium, and hence ‘effective’, temperature (40), which we associate to local transcription levels. Figure 1 shows a schematic of our model, illustrating how a single chromosome within the nucleus is subdivided into monomers with differing levels of activity. Our model classifies each monomer either as ‘active’ or as ‘inactive’ depending on its associated gene density. In our calculations, we assume that known gene densities for human chromosomes, once coarse grained over sufficiently large genomic intervals, should serve as a proxy for transcription-linked activity within those regions. This approximation has the specific virtue that it allows us to pose and answer relatively general questions concerning the role of activity in organizing chromosome positions, but also the attendant shortcoming that cell-type-specific variations in positioning cannot be addressed, at least within this initial version of our model.

Inactive monomers experience stochastic forces of largely Brownian origin, with a scale set by the thermodynamic temperature. Active monomers are assumed to experience a larger magnitude of stochastic forces, of non-equilibrium origin as discussed above. The assumption that chromosomes are in thermal equilibrium corresponds to having all monomers experience the same scale of stochastic forces, irrespective of their gene content, an assumption made unquestioningly in all previous simulation models for chromosomes.

Although our model is motivated by general considerations regarding the importance of non-equilibrium activity, it does differ significantly from other models for active matter. In our model, activity is inhomogeneously distributed across monomeric units (even those belonging to the same chromosome) and confinement plays a crucial role. The spontaneous segregation of particles with differential activity is a fundamental physical consequence of activity, robust across several models for active systems which differ in detail (41–44). We propose that this is the dominant mechanism underlying the compartmentalization of chromosomes based on gene density, terming it ‘activity-based segregation’ in the chromatin context. This study uses well-established polymer models for interphase chromosomes to test this proposal and its implications.

## MATERIALS AND METHODS

We generalize the spherical chromatin domain (SCD) model for chromosomes, described in a previous study (45), to include inhomogeneous activity. We first represent the nucleus as a confining spherical shell, coarse graining each of 23 (human, female) pairs of chromosomes as linear polymer chains composed of spherical monomers representing 1 Mb domains. Such 1 Mb units are basic structural units for chromosome territories (46). We then model confinement effects in non-spherical nuclei, simulating both oblate (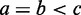) and prolate (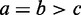) ellipsoids, with *a,b* and *c* representing minor and major axes. A subsequent step is to extend our model to account for preferential interactions of the nuclear envelope with individual monomers.

To incorporate inhomogeneous activity, we use the GeneCards database (47), to compute the number of genes in each 1 Mb monomer unit on each chromosome. Single monomers containing an overall number of genes which fall below a preset cutoff are termed as ‘inactive’, whereas those with a number of genes above the cutoff are termed as ‘active’. Both types of monomers are assigned an effective temperature reflecting their gene content as described in detail below.

Without looping, chromosomes in this and similar models are non-compact, behaving like (ordinary or self-avoiding) random walks at large scales, with the average physical separation between any two monomers increasing as a function of their separation along the chain (genomic distance). However, experimentally, this separation is seen to saturate, leading to compact configurations of individual chromosomes (48). To study how individually more compact chromosomes might segregate, we implement random loop models (49), creating a small number of permanent loops by connecting, with low probability, pairs of monomers chosen at random along the length of each chromosome. Allowing for such compactness, coupled to inhomogeneous activity dictated by gene density yields positioning patterns visually possessing a territorial organization, an observation we further quantify.

### Model for chromosomes in spherical and ellipsoidal nuclei

Our model human chromosomes (22 autosomal pairs and two X chromosomes) are coarse grained as polymer chains made of spheres connected by spring-like (harmonic) links, a coarse graining common to earlier work (45,50–52). We follow the basic methodology and numerical conventions of the SCD model, although our model is simpler in some ways. Each sphere represents a 1 Mb domain of chromosome. The total number of monomers present in the coarse-grained system is 6098. These polymer chains are confined to a hollow spherical region of radius *R*_0_, modeling confinement within a spherical nucleus by the nuclear envelope. Each monomer experiences forces arising from its neighbors which originate in the connecting springs, enforcing polymer connectivity. The spring-based interaction between neighboring monomers (labeled as 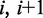, with position coordinates 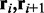) is of the form,
(1)
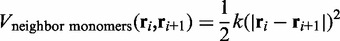

where *k* is a spring constant. Monomers interact with other monomers via a Gaussian interaction (53)
(2)
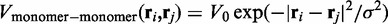



Such an inter-monomer interaction, the Gaussian core potential, arises in the coarse-grained modeling of polymer brushes and originates in the entropic costs of interpenetration of polymers. The effective pair potential at zero separation, *V*_0_, is of order 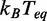, with *k_B_* being the Boltzmann constant. The interaction between each monomer and the confining sphere vanishes if the monomer location falls inside the sphere; outside, it takes the form
(3)
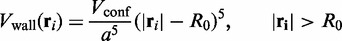

with ‘*a*’, a scale factor discussed below. We choose 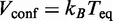. All chromosomes are fairly tightly confined to *R*_0_. The confining potentials are generalized to the ellipsoidal confinement case, taking care to ensure that all points at a common distance from the surface of the ellipsoid experience the same potential. (More details regarding numerical methods for this case are available in Supplementary Data). We study relatively weakly deformed spheres, with aspect ratios 0.3 and 3, scaling the ellipsoid dimensions so as to maintain the same volume as in our simulations with spheres.

### From gene densities to effective temperatures

To incorporate inhomogeneous activity, the gene content of each such 1 Mb region is obtained from the GeneCards database, where the GeneLoc Algorithm (Version 3.09, Nov 2012) is used to create an integrated map of the human genome (47) (Supplementary Figure S1). Single monomers containing a number of genes which fall below a preset cutoff are termed as ‘inactive’ and are characterized by an effective temperature *T_a_* equaling the thermodynamic (physiological) temperature 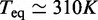. Monomers with a number of genes above the cutoff are termed as ‘active’ and assigned an effective temperature 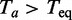 (Supplementary Figure S2). We use mainly two ways of assigning *T_a_* to active monomers: in the first, to verify that inhomogeneities in activity are central, we assign the same temperature 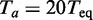 to all monomers, referring to this as the ‘homogeneous activity’ case. In the second, the ‘inhomogeneous activity’ case, we choose the top 5% of monomers in terms of gene content, assigning them 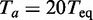, with other monomers retained at *T*_eq_. Note that this assignment is a non-linear one, because only the top 5% of monomers by gene density are taken to be ‘active’ and assigned a larger effective temperature. The second procedure accounts for the following: only a fraction of genes are transcribed in any given cell type, the athermal activity-derived noise in our model arises as a result of a coarse graining both in space and time and there are strong correlations in nucleosome positioning. Specifying that each monomer experiences a local effective temperature dictated by its associated activity, together with assuming that the drag coefficient ζ is a constant (see below), ensures that the steady state of this system is not an equilibrium state.

### Comparisons with experimental data

Experimental data is extracted numerically from Figure 6 [(h): right column] of another study (45) for chromosomes 18 and 19 and from Figure 6 [(g): left column] of the same study for chromosomes 12 and 20; these data originated in two other studies (54,55). These are plotted using open symbols in chromosome-specific colors (see figure captions), when displayed.

### Estimates of effective temperatures

As a measure of the scale and significance of non-equilibrium effects, a recent study finds an up to 10-fold decrease in the diffusion constant of chromosomal loci in live bacteria and yeast upon adenosine triphosphate (ATP) depletion. This accompanies other signatures of the importance of ATP-dependent enzymatic activity in producing stochastic forces that far outweigh those deriving from thermal fluctuations (56). The Einstein relation suggests that effective temperatures are similarly increased over the thermodynamic temperature. Other work stresses that ATP-dependent active sliding, disassembly and sequence-dependent nucleosome positioning, requiring the surmounting of free-energy barriers considerably higher than thermal scales, are crucial to establishing *in vivo-*like nucleosome positioning across biologically relevant timescales (57).

The hydrolysis of a single ATP molecule itself liberates an energy of approximately 20 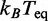, with *k_B_* the Boltzmann constant. In eukaryotes, nucleosomes are positioned (added, removed and slid) by ATP-dependent chromatin remodeling machinery, such as the SWI/SNF, ISW1,CHD/Mi2 and INO80 enzymes, present at large densities in the nucleus (57,58). For human ATP-utilizing chromatin assembly and remodeling factor (ACF), estimates of barriers to nucleosome disruption yield numbers of 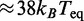, barriers which are surmounted by active processes (59,60). A previous study (57) contains estimates of free energy barriers surmounted by chromatin remodeling machinery that are comparable with the estimates here. Taken together, these suggest that our estimate of active temperature scales, i.e. 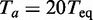 for active monomers, is reasonable. Qualitatively similar results follow even with a smaller spread in effective temperatures; what is crucial for activity-based segregation is ‘inhomogeneity’ in activity, the absolute scale of activity playing a secondary role. Finally, we note that chromosome repositioning within interphase, as when proliferating cells are arrested in senescence or quiescence induced by serum starvation, may involve nucleoplasmic actin and myosin (61–63). Provided only that no large-scale actin scaffolding exists, the action of such localized forces arising out of motor activity can be represented in a manner equivalent to that discussed here, requiring only that it can be modeled as stochastic, athermal and inhomogeneous across chromosomes.

### Simulation methodology

Our numerical evolution of the system of monomers uses an Euler discretization of the Langevin equation describing over-damped motion,
(4)
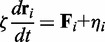

where 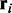 represents the location of the *i^th^* monomer, ζ is a drag coefficient, 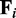 accounts for all non-stochastic forces acting on the monomer and 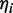 represents stochastic forces arising from both active and thermal fluctuations with scales as described above. Following standard procedures, the noise is assumed Gaussian distributed, with cross-correlations vanishing at all times irrespective of monomer labels. The diagonal correlations, at equal times and for the same monomer, are non-zero and obtained from
(5)
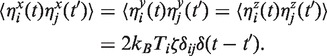



Here, *T_i_* is an ‘effective’ temperature associated to each monomer, reflecting its local level of activity. We represent each of the components of 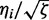 as the product of a Gaussian random number with zero mean and unit variance with the quantity 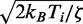. In thermal equilibrium, we have 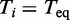 for all monomers, leading to an Einstein relation for individual monomers: 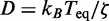. Out of thermal equilibrium, the Einstein relation connecting the drag coefficient and the thermodynamic temperature is violated, the fluctuation–dissipation theorem breaks down and (active) temperatures can differ from monomer to monomer. A detailed discussion of the validity of the concept of active temperature for active polymeric systems is available in another study (40).

### Units and normalization

Following the SCD model, we standardize our parameters against known values, assuming that each domain has diameter 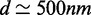, the equilibrium domain separation is 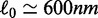 and the nuclear diameter 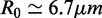. We measure energies in units of 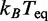, choosing 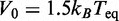. The spring constant is taken to be 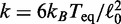. We choose to measure lengths in units of 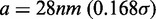, scaling all physical lengths accordingly. We choose units of time (τ) such that 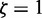 and measure energies in units of 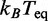. With this choice, τ is then 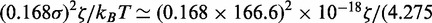

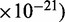
(s). We can approximate the value of ζ appropriate for this calculation from the Stokes relation: 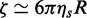 where *R* is the hydrodynamic radius appropriate to the monomer size. Assuming that the appropriate value of the viscosity at such scales is 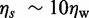, with 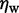 the viscosity of water 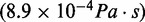, its numerical value is then 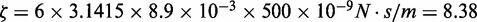

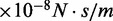
. We now reinsert this to get the unit of time as 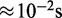; our choice of time-step of 0.01 thus corresponds to real-time evolution by 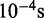. The approximately 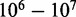 steps taken to attain steady state from a random initial configuration translates to 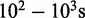 or ∼1–10 min in real time. These are expected to be underestimates, as we do not account for any underlying topological constraints arising from chain crossing (and timescales for their relaxation) although the penetration of monomers is of course penalized energetically. Variations in the drag coefficient arising from differences in effective monomer size between relatively more open euchromatin and more compact heterochromatin regions can also be accounted for, in principle, but are expected to be small as a consequence of the relatively weak dependence of the friction coefficient on the monomer size.

### Analysis

Our simulations are run for at least 10^7^ time steps, with around 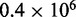 steps discarded to ensure adequate equilibration. All data are averaged over at least 10^6^ independent measurements. We verified that the same steady state properties were achieved irrespective of initial (random) configuration. As the probability of finding a chromosome at a radial separation **r** from the origin depends only on the modulus of **r**, i.e*.* 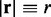, we calculate the probability of finding a monomer belonging to a specific model chromosome at a radial distance from the origin for each chromosome. We calculate the quantity 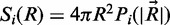, where 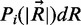 is proportional to the probability of finding a monomer of chromosome *i* at a radial vector 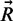 from the origin. For a uniform distribution, 
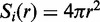
, giving the quadratic rise exhibited in the figures if activity is uniform. We compute 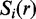 for every model chromosome. We measure activity in successive radial shells by performing a configurational average over the effective temperature of every monomer in that shell. From these, we extract a quantity similar to *S*(*r*), but normalized by 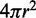, so that the quantity plotted in the cut-away sphere representation simply represents the activity at radial distance *r*, rather than the net activity in that whole shell. To visually examine configurations, we color-coded monomers belonging to individual chromosomes. In our simulations of the ellipsoidal case, we compute similar quantities, scaling our distribution functions so that points equidistant from the boundary are averaged over, to obtain densities as a function of an effective radial coordinate. We also calculate the evolution of averaged distributions, computed at a sequence of times and averaged over a number of initial conditions initiated from states where the wall effect is turned off. Details regarding the benchmarking of values for this case are available in Supplementary Data, (‘Active Interactions with the Nuclear Envelope’ section) and procedures are described in Supplementary Data (‘Positioning Intermediates Obtained as NE Interactions are Turned On’ section).

### Combining random loop models with inhomogeneous activity

Several models for chromosome conformations at large scales ensure compactness of chromosome configurations by permitting a small fraction of chromosomal subunits to be connected, even if they are well-separated along the chromosome length (48,49,64); other models assign a similarly compact non-equilibrium fractal globule-like structure to each chromosome (65). Random loop models, e.g. previous studies (66,67), are a class of such models constructed by allowing pairs of segments of the chromosome, well separated in sequence space, to associate. Here, we implement the random loop model, connecting pairs of randomly chosen monomers along the length of each chromosome with low probability. We note that 3C and 4C methods provide evidence for loops of sizes of several tens of Mb (68); loop sizes falling within our 1 Mb discretization are coarse grained into the interaction potential. This connection is assumed to be permanent once formed. It is physically represented by springs with a spring constant which is 10 times larger than for other monomers connected to their neighbors along the same chain. We have studied three values of the looping probability: 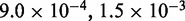 and 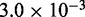. (As a measure of the number of loops, these choices yield 424, 712 and 1404 loops within a specific realization for the 6098 monomers we consider). Chromosomes with a small density of such random loops are equilibrated and their configurational properties calculated.

### Quantifying chromosome territory formation

About the centre of each monomer, we construct a sphere of radius *R*_sphere_, which we are free to vary. The sphere, if large enough, contains the centers of multiple monomers. For monomers belonging to the same chromosome as the initially selected monomer, we assign +1, while for monomers belonging to different chromosomes, we assign −1, summing these over all monomers whose centers are contained within the sphere. We then plot this summed quantity, which we call 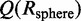 averaged over all monomers belonging to all chromosomes (Figure 8B).

## RESULTS

From an ensemble of steady-state configurations, we evaluate a number of structural parameters and distribution functions for our model chromosomes. We generate model predictions for all chromosomes but concentrate here on the pairs 18/19 (Chromosome 19: 62.03 genes/Mb and 60 Mb size, Chromosome 18: 18.64 genes/Mb and 78 Mb size) and 12/20 (Chromosome 12: 30.92 genes/Mb and 134 Mb size, Chromosome 20: 29.71 genes/Mb and 63 Mb size) for specificity. Note that Chromosomes 18 and 19 are similar in size but differ in gene density, whereas chromosomes 12 and 20 differ in size but have comparable gene densities, making them ideal candidates to test for segregation by gene density. We also measure local activity distributions in steady state at radial distance *R*, by averaging active temperatures associated with all monomers in a small range about *R*. In our simulations of the ellipsoidal case, we compute similar quantities, scaling our distribution functions to obtain densities as a function of an effective radial coordinate. Figures 3–6 and Supplementary Figure S3 contain results for the model without loops, whereas Figures 7 and 8 are for the general random loop model with inhomogeneous activity.

**Figure 2.**
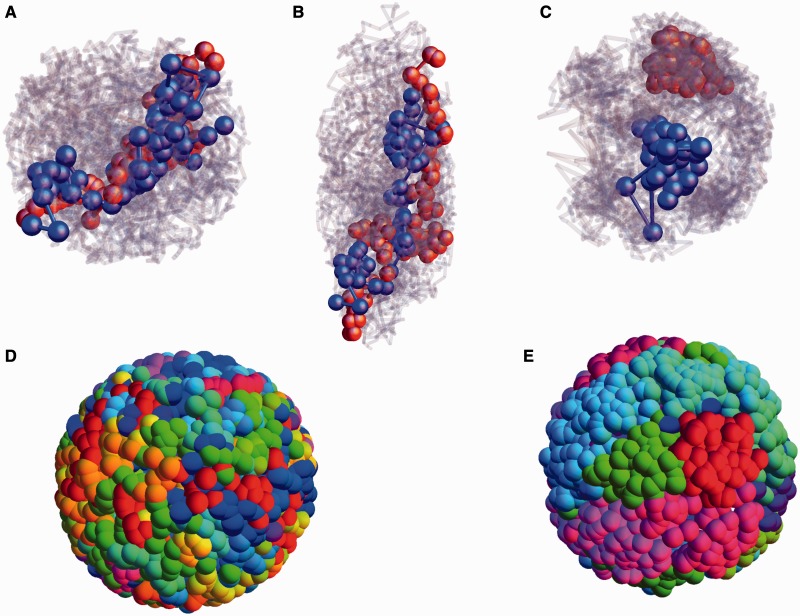
Configurational snapshot showing positions of simulated human chromosome 18 (red) and chromosome 19 (blue) for our model in the background of other chromosomes (gray), for (**A**) and (**C**), spherical nuclei and (**B**), a prolate ellipsoidal nucleus. The surface configuration of monomers, color coded by chromosome, are shown in (**D**) and (**E**). Chromosomes contain no permanent loops in (A), (B) and (D), whereas the configurations in (C) and (E) represent a snapshot of the more compact configurations obtained by allowing for a fixed, small density of loops of random sizes. The nuclear envelope is represented by a repulsive, short-ranged potential, confining chromosomes to a given geometry in all these cases. These simulations in (A) and (B) represent thermal equilibrium, with chromosomes displaying substantial intermingling. In contrast, the configurations in (C), for a model of compact chromosomes, represent the non-equilibrium case discussed in this article, reflecting the ‘activity-based segregation’ of chromosomes, concomitant with the formation of chromosome territories. The more gene-dense chromosome 19 occupies a more interior position than does the gene-poor chromosome 18 in (C). (D) is the non-equilibrium case with no loops. The contrast between (D) and (E) illustrates how activity-based segregation, a feature of both these snapshots, is insufficient to generate chromosome territories on its own. In (E), for a model of compact chromosomes constructed using the random loop model, the combination of such compactness and activity-based segregation yields configurational snapshots which clearly show evidence for a territorial organization.

**Figure 3.**
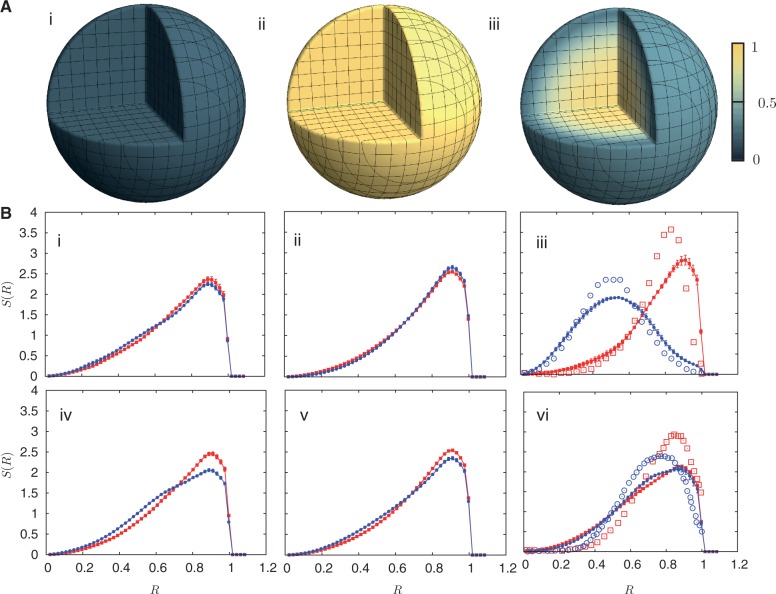
Activity distributions and *S*(*R*) for specified chromosome pairs in a spherical nucleus**.** (**A**) (i)–(iii) show a cut-away section illustrating the time-averaged local activity measured in units of the time-averaged local monomer active temperature, color coded with activity strength and normalized to the range (0:1). The color code correspond to the interval between the thermodynamic temperature *T*_eq_ and the active temperature assigned to monomers with a high density of active genes, 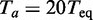. The darkest shades represent *T*_eq_, whereas the lightest shades represent *T_a_*. (A) (i) represents the thermal equilibrium case, whereas (A) (ii) represents the case where all monomers are assigned a uniform high activity. (A) (iii) shows the case for non-uniform activity. Note that the distribution of activity is unstructured in cases (i) and (ii), being uniformly low in the first case and uniformly high in the second, while it is structured in case (iii), with activity enhanced towards the center. While the data in (i)–(iii) are averaged over all chromosomes, data for the chromosome pairs 18 (red-filled squares) and 19 [blue-filled circles; (**B**) (i)–(iii)] as well as for 12 (red-filled symbols) and 20 (blue-filled symbols) [B (iv)–(vi)], are shown corresponding to the activity distributions above them. Note that for the uniform activity case, these chromosome pairs are distributed uniformly, with *S*(*R*) quadratically increasing toward the nuclear periphery. (B) (iii) and (vi) show these distributions in the case where activity is non-uniform, illustrating that these distributions are non-trivially structured, being enhanced toward the nuclear interior in the case of the more active chromosome. Along with the simulation data in (B) (iii) and (vi) (filled symbols), we also show open symbols in the same chromosome-specific color representing the experimental data displayed in another study (45) for these chromosomes. Error bars indicated refer to standard deviations.

**Figure 4.**
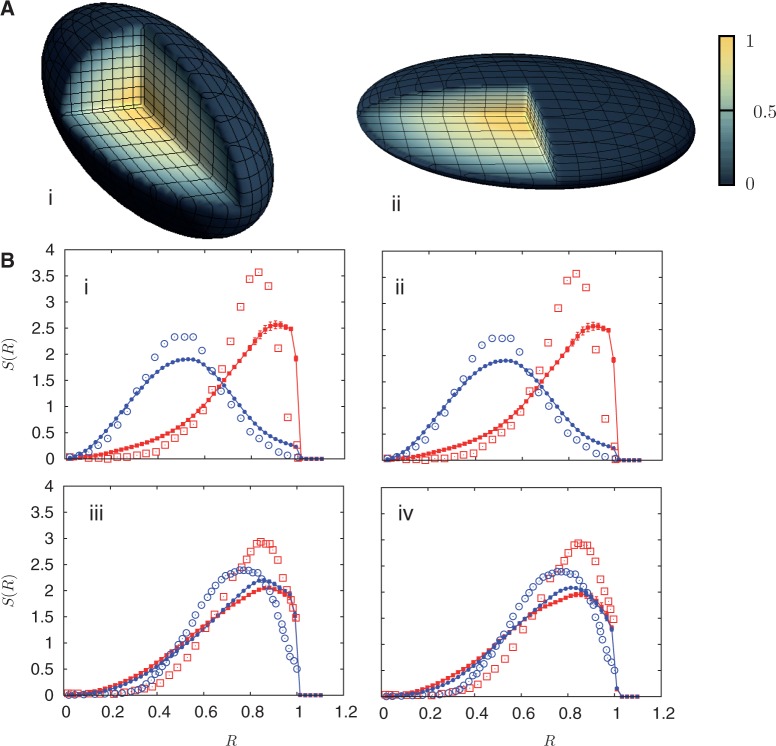
Activity distributions and *S*(*R*) for specified chromosome pairs in an ellipsoidal nucleus. (**A**) (i) and (ii) show a cut-away section representing the time-averaged local activity, as discussed in the caption of Figure 2 and in the main text, for the case of a prolate ellipsoid in A (i) and an oblate ellipsoid in A (ii). These are shown for the case of non-uniform activity, but where chromosomes uniformly experience a passive interaction with the nuclear envelope, acting through a confining short-ranged potential. Data for the chromosome pairs 18 (red-filled squares) and 19 (blue-filled circles) [(B) (i) and (ii)] as well as for 12 (red-filled symbols) and 20 (blue-filled symbols) [(B) (iii) and (iv)] are shown. The distributions are to be compared with the distributions in Figure 2B (iii) and (vi) and indicate that geometrical confinement does not qualitatively alter the positioning of our model chromosomes. We also show the experimental data displayed inanother study (45) as open symbols in the corresponding chromosome-specific colors.

**Figure 5.**
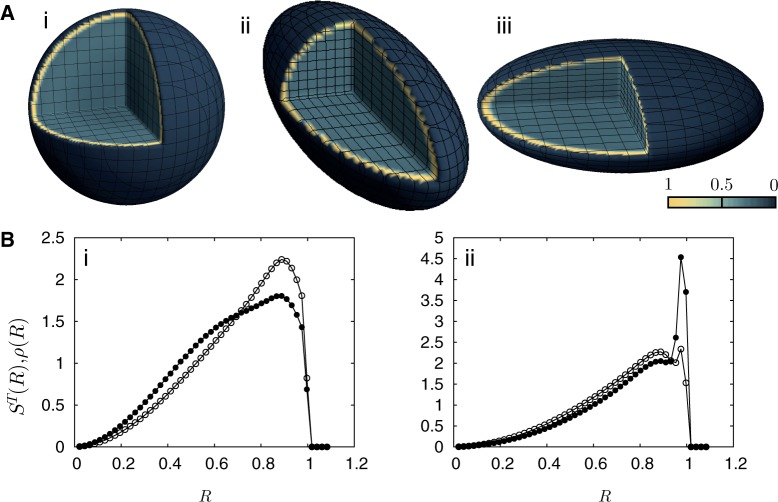
Activity distributions, total densities and gene densities in an ellipsoidal nucleus allowing for specific interactions with the NE. (**A**) (i)–(iii) show a cut-away sphere, prolate ellipsoid and oblate ellipsoid displaying the time-averaged local activity measured in units of the thermodynamic temperature, color coded with activity strength as indicated, following the conventions of Figures 2 and 3. The confinement exerted by the nuclear envelope is now taken to be active, acting specifically on those monomers at a higher effective temperature interacting with them through a short-ranged attractive potential whose minimum is at the boundaries of the nuclear envelope. Note that the earlier described segregation of activity is now substantially modified, with the distribution of more active monomers peaking towards the periphery as opposed to its more central location in Figures 2 and 3. (**B**) (i) shows the total density [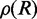: open circles] and the gene density [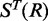: filled circles] for the case of inhomogeneous activity but with a passive interaction with the nuclear envelope, whereas (ii) shows these quantities in the presence of a selective interaction of active monomers with the inner surface of the simulated nucleus, both for a spherical nucleus. Note that only a small fraction (5%) of monomers are active and therefore feel the attraction due to the boundary. Such a marginal effect has strong consequences for positioning, inverting the conventional (active/euchromatin inside, inactive/heterochromatin outside) arrangement of chromosomes.

**Figure 6.**
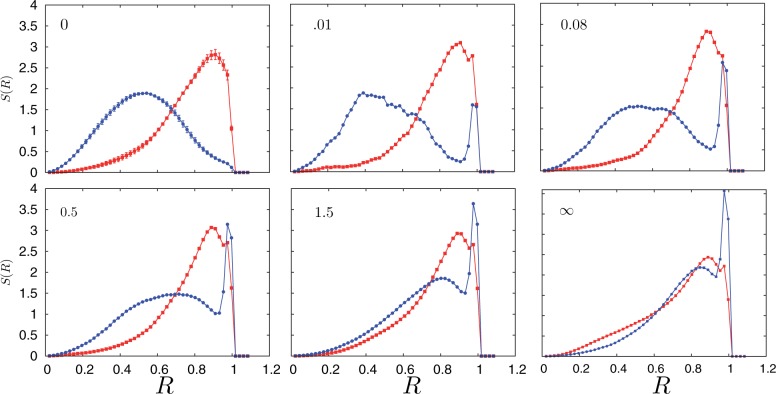
Evolution of the positional distribution of chromosomes 18 (red squares) and 19 (blue circles), shown as a function of time in sequence as indicated, following the switching on of the active interaction with the nuclear envelope in a simulated spherical nucleus. Initial configurations are drawn from a steady-state ensemble prepared by taking the nuclear confinement to be passive. Time is measured in units of millions of simulation timesteps as shown in each subgraph and 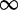 denotes the steady state. This sequence illustrates how sequences intermediate between steady state ones can be attained and possibly stabilized by other processes, not included in the present description, yielding a broad variety of distributions in *S*(*R*).

**Figure 7.**
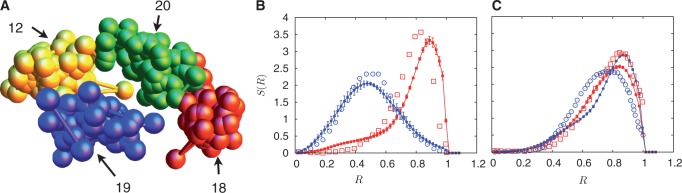
Emergence of chromosome territories and *S*(*R*) for specified chromosome pairs in a spherical nucleus assuming a random loop model. In (**A**), we show instantaneous configurations of chromosomes 18, 19, 12 and 20 for the random loop model with inhomogeneous activity, providing evidence for spatial separation and considerably reduced intermingling. In (**B**), the probability distribution of chromosomes 18 (red-filled squares) and 19 (blue-filled circles) in the compact case is shown, indicating that the activity-based segregation studied earlier continues to be seen in this case. The looping probability chosen is 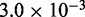, yielding ∼1400 loops for the 6098 monomers we consider. As before, probability distributions for chromosomes with similar gene densities, chromosomes 12 (red-filled squares) and 20 (blue-filled circles) in (**C**) show no segregation. Again, as before, the corresponding experimental data of another study (45) is shown as open symbols of the same color.

**Figure 8.**
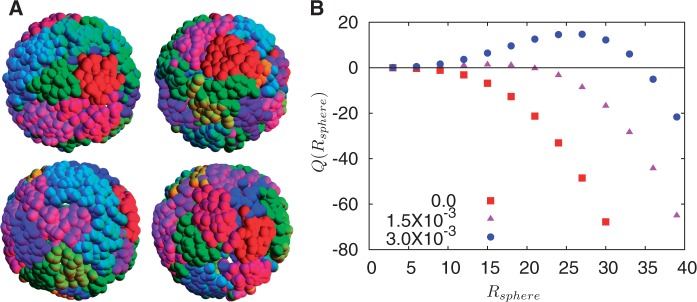
Robustness of chromosome territories and their quantification. (**A**) Four steady-state surface configurations of monomers belonging to different chromosomes, color coded by chromosome, with each configuration obtained using a different initialization. These illustrate, pictorially, the observation that chromosome territory formation is not a property of a specially chosen initial condition, but arises naturally and in an emergent way from the twin requirements of activity-based segregation and compact chromosome configurations, in this case enforced through the random loop model for chromosomes. In (**B**), we show 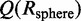, a quantity constructed by situating around each monomer a sphere of radius *R*_sphere_, counting the number of monomers belonging to the same chromosome weighted by the quantity +1 and the number of monomers belonging to different chromosomes, weighted by –1. This quantity provides an indication of whether chromosomes are organized territorially and in a non-overlapping manner. We show results for three values of the looping probability: 0, 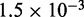 and 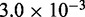. As the number of random loops is increased, leading to more compact chromosome configurations, the tendency for overlap is reduced, further enhancing the tendency to separate which is a consequence of activity-induced segregation.

### Our model predicts chromosome positioning across a variety of conditions

The sequence of figures in Figure 2 (A–C), exhibit illustrative snapshots of chromosomes in our model under a variety of different conditions. We model different nuclear shapes, vary the large-scale configurations of individual chromosomes as well as study both equilibrium and non-equilibrium situations. Figure 2 (A–C) shows chromosomes 18 (red) and 19 (blue) within the background of other chromosomes, shown in grayscale background. These are displayed in Figure 2A, for a simulated spherical nucleus and in Figure 2B for a simulated prolate ellipsoidal nucleus. The simulations in (A) and (B) are for thermal equilibrium, with 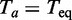 for all monomers.

In contrast to these, Figure 2C shows configurations for our non-equilibrium model with inhomogeneous activity. Here, following the random loop model, we assign individual chromosomes a fixed, small density of loops of arbitrary sizes, thus ensuring that the resulting chromosome configurations are compact. The results for this case reflect ‘activity-based segregation’, in which bulk intermingling is considerably suppressed, a territorial organization emerges and the gene-dense chromosome 19 occupies a more interior position than does the gene-poor chromosome 18. In addition, Figure 2D and E display the surface configuration of monomers, both for the inhomogeneous activity case. A configurational snapshot with non-compact chromosome configurations (no loops) is shown in Figure 2D.

Figure 2E provides a snapshot for the case of compact chromosome configurations, generated through the random loop model described above. As can be seen by comparing Figure 2D–E, activity-based segregation alone does not give rise to chromosome territories if there is no mechanism to create compact chromosome configurations. Inducing such compactness, by the expedient of allowing for a small density of random connections along each chromosome, allows chromosomes to segregate by activity as well as reduces intermingling. The surface configurations of Figure 2E provide striking visual evidence for a territorial organization of chromosomes in the random loop model coupled to inhomogenous activity.

### Chromosome positioning is unstructured if non-equilibrium activity is absent or uniform

Figure 3A [(i)–(iii)] shows cut-away spheres displaying the time-averaged local activity at different points within the spherical nucleus. Activity is measured in units of the thermodynamic temperature, color coded with magnitude and further scaled to the interval (0:1) as shown. (The color code thus corresponds to the interval between *T*_eq_ and 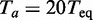, with the darkest shades representing *T*_eq_ and the lightest shades *T*_a_). Given the relationship we assume between activity and gene density, a uniform color indicates that gene densities are uniform. Figure 3A (i) represents such distributions in thermal equilibrium, whereas Figure 3A (ii) represents the case where all monomers are assigned a uniformly high activity temperature of 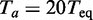. The distribution of activity is unstructured in both cases; it is uniformly low in the first case and uniformly high in the second.

Data for specific chromosome pairs are shown in Figure 3B (i) and (ii) (for chromosomes 18 and 19; blue and red, respectively) as well as (iv) and (v) (for chromosomes 12 and 20; blue and red, respectively), corresponding to the activity distributions in Figure 3A (i) and (ii) directly above them. Both show that chromosomes are distributed uniformly with respect to their gene density, with *S*(*R*) showing the expected quadratic increase toward the nuclear periphery. The marginal differences between the distributions for the constant activity case reflect both interactions and the poly-dispersity of chromosomes; they also include the effects of entropic interactions between chromosomes. Such effects are overwhelmed once inhomogeneous activity, to be discussed next, is incorporated into the model.

### The gene density-based radial segregation of chromosomes is a robust consequence of inhomogeneous activity

Figure 3A (iii) summarizes a major result of our work: the non-uniform spatial distribution of local activity which obtains when active temperatures, reflecting the strength of local non-equilibrium force fluctuations, are assigned in a manner proportional to gene density. The distribution of activity differs strikingly from that shown in Figure 3A (i) and (ii). Activity distributions in Figure 3A (iii), reflecting gene densities, peak towards the center and are suppressed toward the periphery, indicating gene density-based segregation. In comparison with the case for uniform activity, (shown in Figure 3B (i) and (iv) and Figure 3B (ii) and (v), as discussed previously), Figure 3B (iii) shows *S*(*R*) for the inhomogeneous activity case. Experimental data for *S*(*R*) associated to chromosomes 18 and 19 in human lymphocytes is also plotted for comparison. These figures, in distinct contrast to those of Figure 3A (i)–(ii), clarify that inhomogeneous activity drives the segregation of chromosomes, with the more gene-dense chromosomes found toward the center of the nucleus. Our model, despite its simplicity, yields results that quantitatively reproduce the experimental magnitude of these relative shifts. *S*(*R*) plots for all chromosomes are available in Supplementary Data (Supplementary Figure S3) for the model without loops but with non-equilibrium activity),

### Purely geometrical confinement arising from nuclear shape is a relatively minor determinant of positioning

Figure 4A (i) and (ii) shows cut-away spheroids, both prolate as in Figure 4A (i) as well as oblate in Figure 4A (i), exhibiting the time-averaged local activity. Figure 4B (i)–(iv) showing *S*(*R*) for the chromosome pairs 18 (red) and 19 (blue) as well as for chromosomes 12 (red) and 20 (blue), in the case of purely passive (geometrical) confinement, but accounting for inhomogeneous activity. These figures resemble those of Figure 3A (iii) and 3B (iii) and (vi) both qualitatively and quantitatively. We also see no substantial difference in the relative positioning of chromosomes vis–a-vis the spherical case. This suggests that effects arising from purely geometric confinement are likely to be intrinsically small and that nuclear shape, acting on its own, may not be a strong determinant of chromosome positioning.

### Allowing specific interactions of even a small number of monomers with the nuclear envelope strongly modulates positioning

Genome organization is often tissue specific, e.g. mouse chromosome 5 is more centrally positioned in liver cell nuclei but not in nuclei from lung tissue, where it is seen more peripherally (69). Activity-based segregation favors the conventional arrangement, in which gene-poor heterochromatin is found toward the nuclear periphery, whereas gene-rich euchromatin regions are found toward the center of the nucleus. However, arrangements inverted with respect to the conventional one have been seen in rod cells from the retina of mice, most likely reflecting adaptation to a nocturnal lifestyle (16). In these cells, all genes, irrespective of their transcriptional status, were found localized toward the nuclear periphery, in most cases juxtaposed to the nuclear lamina (16). Recent experiments suggest that inversion in this case is a consequence of the absence of specific lamins and proteins (lamin A/C and lamin B receptor), which normally act to tether heterochromatin to the NE (70). Chromatin interactions with the nuclear lamina, *en route* to lineage commitment and terminal differentiation, have been suggested to play a central role in genome reorganization (27,28). Changes in these interactions may possibly limit the rate at which both cellular differentiation and reprograming occur (71–73).

The approximations we have made preclude cell type-specific analyses, but our model permits us to identify a fraction of monomers which can then interact preferentially with the NE. We can then ask the following question: can a suitably chosen fraction lead to stable inverted arrangements as terminal states? (We study this specific question because the conventional and inverted arrangements are two extreme limits of non-random radial distributions ordered by gene density; if our model can recover these extremes, reproducing any radial positioning pattern intermediate between them should be straightforward). To study this, we allow the NE to act specifically on ‘active’ monomers through a short-ranged attractive potential, with a minimum proximate to the NE. Inactive monomers experience the usual repulsive confining potential. This choice is the simplest we can make; only requiring estimates for the strength and range of the NE-induced interaction, discussed in ‘Simulation Procedures’.

Adding such an interaction dramatically alters positioning patterns. Figure 5A (i)–(iii), show activity distributions corresponding to spherical, prolate and oblate nuclei, illustrating that activity now peaks in a narrow band toward the nuclear periphery, representing an increased density of genes in proximity to the NE. Below these, we show, in Figure 5B (i) and (ii), the total density (open circles), as measured in our coarse-grained 1 Mb units, as a function of the radial coordinate *R* as well as the gene density (filled circles), for the case of inhomogeneous activity. There is no selective interaction with the NE in the data shown in Figure 5B (i), whereas the effects of such an active interaction is included in the results of Figure 5B (ii). Although both data show a quadratic rise close to the origin, the number of genes close to the NE is substantially enhanced in Figure 5B (ii).

Figures 4 and 5 thus jointly illustrate the following: purely geometrical effects arising from nuclear shape are weak, at least in our model. However, making the nuclear envelope selectively attractive to a small fraction of monomers can drastically alter positioning patterns, even inverting the conventional arrangement based on gene density. Thus, the competition between bulk (activity-based segregation) and surface (selective interactions of monomers with the NE) e.g. see Ref. (70), provides a route to radial positioning schemes different from the conventional one, even though only a relatively small fraction of monomers—5% in this case—selectively interacts with the NE.

### The dynamical evolution of chromosome positions upon perturbation can be tracked in our model

In our model, we can also investigate questions of more dynamical significance, such as the intermediate distributions obtained between two terminal ones, following the initiation of a perturbation. We have investigated a specific perturbation, the reorganization of chromosome positions in response to a NE perturbation acting as described above, which leads to inverted arrangements as terminal steady states. In Figure 6, we show the evolution of *S*(*R*) as a sequence of snapshots of configurations of chromosomes 18 and 19, as tracked from the time instant where the NE interaction is switched on. We show, in temporal sequence, the evolution of *S*(*R*) captured in units of time-steps measured in millions of time-steps. The terminal figure corresponds to the final steady state distribution. Within the assumptions we make, our model can thus provide predictions for distribution functions at intermediate times, arising out of a perturbation.

### Chromosome territories emerge from the combination of compact configurations for individual chromosomes and activity-based segregation

Activity is a more potent driving force than entropy, as associated characteristic energy scales far exceed those set by physiological temperatures. One might guess that individually more compact configurations, however they might be generated, could be more effectively segregated by activity than by purely entropic means. Recall that in Figure 2D, we displayed a configurational snapshot of monomers on the nuclear surface, with each chromosome colored differently, for the case of non-compact chromosomes with inhomogeneous activity. The relatively random distribution of colors on the surface indicates that chromosomes are intermingled. No visible tendency toward territory formation is seen. In Figure 2E, a similar snapshot is provided for the non-equilibrium case but initialized from an ensemble of more compact chromosome configurations, created via the random loop model. Remarkably, such configurational snapshots show consistent and strong evidence for a territorial organization, visually resembling the standard 3d-FISH (fluorescence *in situ* hybridization) images used to infer a territorial organization of chromosomes.

This is further examined in Figure 7A, where we show configurations of chromosomes 18, 19, 12 and 20 for the random loop model with inhomogeneous activity, providing evidence for spatial separation and considerably reduced intermingling of chromosome segments in the bulk. The looping probability chosen is 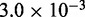. In Figure 7B, we display the probability distribution *S*(*R*) for chromosomes 18 and 19 (blue and red, respectively) in the compact case, indicating that the activity-based segregation studied earlier is a phenomenon robust to allowing for such drastic changes to the configurational properties of individual chromosomes. As before, probability distributions for chromosomes with similar gene densities, as chromosomes 12 and 20 (blue and red, respectively in Figure 7C), show no segregation. Allowing for compactness substantially improves the agreement between simulations and experimental data, as can be seen by comparing Figure 3B (iii) and (vi) with Figure 7B and C.

### Territory formation is a robust feature of our model

To what extent is such territorial organization a robust feature of our model? To address this, we compare the final chromosome configurations which result from a number of separate random initializations, to check that such a territorial organization is manifest across them. Figure 8A shows four such configurations, obtained in steady state by simulating from independent initial states in which the monomers belonging to each chromosome are positioned at random. Each color represents a separate chromosome. Note that each of these configurations separately shows a territorial organization of chromosomes, although these configurations are not identical, illustrating that such organization is a natural property of the steady state of this model. In particular, no fine tuning or specific choices of initial conditions is required, as in some earlier work (45). We note that recent polymer-based models for territoriality invoke the topological properties of ring polymers, assigning a crucial role to the inability of polymeric DNA to cross at intersections (74). However, the relevance of such topological constraints is hard to assess because in contrast to *in vitro* polymer systems for which such constraints are effectively absolute, DNA topology-modulating enzymes such as type-II topoisomerases, specifically capable of relaxing such constraints, are present in large numbers within the nucleus.

To further quantify this visual result, we evaluate the quantity 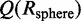 discussed in ‘Materials and Methods’ section. Figure 8B shows that for more compact chromosome configurations induced through a larger number of random loops, the tendency to overlap is reduced, further enhancing the tendency to separate, which is a consequence of activity-induced segregation. Note that for large *R*, this quantity is negative, decreasing further as *R*_sphere_ is increased. This reflects the fact that at large enough scales, the sphere drawn about each monomer contains multiple monomers belonging to different chromosomes. If there are no loops, substantial intermingling results and this probe of territoriality remains negative, reflecting the presence of monomers belonging to other chromosomes in the near vicinity of a monomer belonging to a given one. This is the case for loop probability 0. The peak in 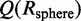 can tentatively be assigned to an averaged territory size, although the physical interpretation of this quantity is complicated by the fact that it is averaged over chromosomes of different sizes.

## DISCUSSION

The higher order organization of chromatin subtly modulates gene expression programs via epigenetics, linking local gene expression with the substantially larger physical scales of chromatin structuring and chromosome positioning (75–79). Here, we showed how one form of such higher order organization, the robust radial segregation of chromosomes based on gene density, could be obtained within a relatively simple model. Previous work implicitly assumed chromosomes to be in thermal equilibrium. However, all available evidence indicates that stochastic forces associated to ATP-consuming (active) processes involved in chromatin remodeling and transcription dominate over thermal forces (56,57). Arguing that the inhomogeneous distribution of genes on each chromosome should lead to a similar inhomogeneity in the non-thermal (active) noise experienced by chromosomal segments with differing levels of active and inactive genes, we proposed that the relatively recently understood physical phenomenon of segregation in systems of active particles with varying motility might underly gene density-based chromosome segregation.

Assuming that gene density, once coarse grained over a 1 Mb region, could be taken as roughly representing the magnitude of transcription-linked activity within that region, we showed that segregation of the appropriate magnitude could be reproduced in a relatively simple model for interphase chromosomes. The predictions of this model include a detailed study of positioning of all human chromosomes, illustrating how chromosomes are differentially distributed as a function of their gene density. For the case of chromosomes 18 and 19 as well as of 12 and 20, our predictions compare favorably with experimental data on chromosome positioning in near-spherical human lymphocyte nuclei.

While purely geometrical effects appear weak *a priori*, the fact that the sphere is simply the geometrical figure with the smallest surface area at given volume suggests that surface-specific interactions are likely enhanced in non-spherical geometries, such as in the fairly flat cells in which size-dependent segregation was seen earlier. Adding a selective interaction drawing active monomers toward the nuclear envelope altered positioning, stabilizing distributions inverted with respect to the conventional one. Thus, we demonstrated that two extreme limits of gene density-dependent radial distributions could be generated, stably and reproducibly, with minimal assumptions.

We found that combining mechanisms ensuring more compact chromosomes with the intrinsic, activity-derived tendency of chromosomes to segregate led directly to configurations closely resembling the territorial organization seen in the FISH imaging of individually painted chromosomes. This striking result, obtained without any requirement for fine tuning leads us to suggest that non-equilibrium activity may be largely responsible for the territorial organization of chromosomes. Allowing for the formation of a small number of loops thus generating more compact configurations of individual chromosomes maintained activity-based segregation, while also yielding closer agreement with the experimental *S*(*R*).

A central requirement of our model is that ‘local’ measures of the magnitude of stochastic non-equilibrium forces experienced by different sections of individual chromosomes should correlate to local ATP-consuming enzymatic activity. When gene density-based segregation is initially established, likely in early G1 where chromosome movements are largest (80), we expect that the scale of such stochastic forces should decrease in a quantifiable manner between nuclear center and periphery, as Figure 3A (iii) indicates. DNA-repair machinery is ATP consuming and we consider it plausible that the reversible chromosome territory movements observed during DNA damage repair reflect varying levels of chromosome-specific internal repair-related activity (81). Correlating chromosome-specific levels of damage to alterations in positions should test our hypothesis that the scale of active forces experienced by individual chromosomes is related to the positions they occupy relative to the nuclear center. In addition to the situations listed above, chromatin remodeling performs a central epigenetic regulatory function in replication, apoptosis and development, suggesting that at least some fraction of large-scale chromosome movements in these situations may reflect inhomogeneous activity of the sort we discuss here. Finally, *in vitro* studies of positioning patterns involving artificial chromosomes, where activity can be induced or suppressed in a controlled manner, may provide tests of our hypothesis that activity controls large-scale positioning.

## CONCLUSION

Radial gene density-based chromosome organization and territory formation may represent foundational principles of spatial genome structuring which are common to multiple cell types (3). The mechanism we propose for these is attractive because it is general and robust. Our work also clarifies why previous attempts to understand segregation from purely equilibrium considerations were incomplete. Given the generality of these ideas, we expect that they may be applicable to large-scale DNA organization in lesser eukaryotes as well as in bacteria (82).

In Meaburn and Misteli’s ‘self-organisation model’ for chromosome positioning, ‘the position of each chromosome is largely determined by the activity of all its genes; that is, the number and pattern of active and silent genes on a given chromosome' (2). Here, we link such patterns of gene activity directly to physical positioning *via* inhomogeneous activity, suggesting a specific physical mechanism for such self-organization.

The importance of non-equilibrium activity for theoretical descriptions of chromatin and the demonstration that gene density-based segregation and the formation of territories have a common and robust underlying origin in our model system via ‘activity-based segregation’, are the central results of our work. We suggest that activity-based segregation might provide a generic initial template for local physical and biochemical events acting to further stabilize and optimize positioning. These include, but are not limited to, the effects of nuclear shape at larger aspect ratios, selective interactions with the nuclear envelope and associated chromatin binding proteins, potential transient interactions with localized dynamic clusters of nuclear myosin motors and actin as well as, importantly, the spatial clustering of active genomic regions associated to the intermingling of looped chromosomal segments in inter-chromatin domains or at transcription factories (1,83). Each of these would supplement such a generic template with incremental layers of cell-type-specific organization. Generalizing our model to incorporate these effects should yield more complex proximity patterns. Comparing such *in silico* predictions with experimental data at increasing levels of microscopic resolution can be expected to yield useful insights into the multiple determinants of chromosome positioning.

## SUPPLEMENTARY DATA

Supplementary Data are available at NAR Online, including [47,84].

## FUNDING

Travel and computational support from the TIFR Centre for Interdisciplinary Science (India) (to N.G.); Department of Atomic Energy—Science Research Council Outstanding Researcher Fellowship (to G.I.M.); support for a sabbatical appointment from the National University of Singapore, Singapore (to G.I.M.). Funding for open access charge: Institutional funds.

*Conflict of interest statement.* None declared.

## Supplementary Material

Supplementary Data
