# Supplementary material for: Chromosome positioning from activity-based segregation
Source: Nucleic Acids Res. 2014 Jan 22;42(7):4145–59. doi: 10.1093/nar/gkt1417 (PMC3985638; doi:10.1093/nar/gkt1417)
Supplement: Supplementary Data [file supp_42_7_4145__index.html]

Chromosome positioning from activity-based segregation — Chromosome positioning from activity-based segregation — Supplementary Data 

# Chromosome positioning from activity-based segregation

## Supplementary Data

files

**Files in this Data Supplement:**

- Supplementary Data - pdf file
