# Supplementary material for: Chromosome positioning from activity-based segregation
Source: Nucleic Acids Res. 2014 Jan 22;42(7):4145–59. doi: 10.1093/nar/gkt1417 (PMC3985638; doi:10.1093/nar/gkt1417)
Supplement: Supplementary Data [file supp_gkt1417_nar-03102-n-2013-File013.pdf]

Online Supplementary Information

Chromosome Positioning from Activity-based Segregation

Nirmalendu Ganai, Surajit Sengupta and Gautam I Menon

I. Gene density and Activity Assignments

The monomer-wise count of the number of genes corresponding to each chromosome in our calculation is provided in Fig. S1. The horizontal line in each figure indicates the cutoff value which we use to impose our threshold for counting an individual monomer as being active. Fig. S2 shows the histogram of gene densities, together with our 5% cutoff. Note that there are relatively few monomers with high gene density, with the bulk of the weight of the histogram concentrating in the regime of low gene density.

II. Simulating Oblate and Prolate Nucleus Geometries

Here we describe our simulation methodology for chromosomes in non-spherical geometries, later generalizing this to the case in which the confinement provided by the nuclear envelope has an additional attractive component for active monomers. We first treat the case in which all monomers interact equally with the confining wall. For the spherical case, evaluating $(|\mathbf{r}_i| - R_0)^5$ for each monomer is straightforward, since the closest distance to the sphere boundary lies along the radial direction provided by measuring $\mathbf{r}_i$ from the centre of the sphere. This does not hold for the aspherical situation, requiring that we compute the *shortest* distance of a monomer from the wall independently. The distance of the ith monomer from the origin, located through its Cartesian coordinates $\vec{p_i} = (x_i, y_i, z_i)$ is

$$r_i = \sqrt{x_i^2 + y_i^2 + z_i^2}$$

To measure the closest distance of the monomer from the wall, we assume that the (prolate or oblate) ellipsoid is centred at the origin and is parametrized as

$$x(\theta, \phi) = a \cos\theta \sin\phi$$
$$y(\theta, \phi) = a \cos\theta \cos\phi$$
$$z(\theta, \phi) = c \sin\theta$$

with $a < c$ and $a > c$ the prolate and oblate cases respectively. This yields the equation

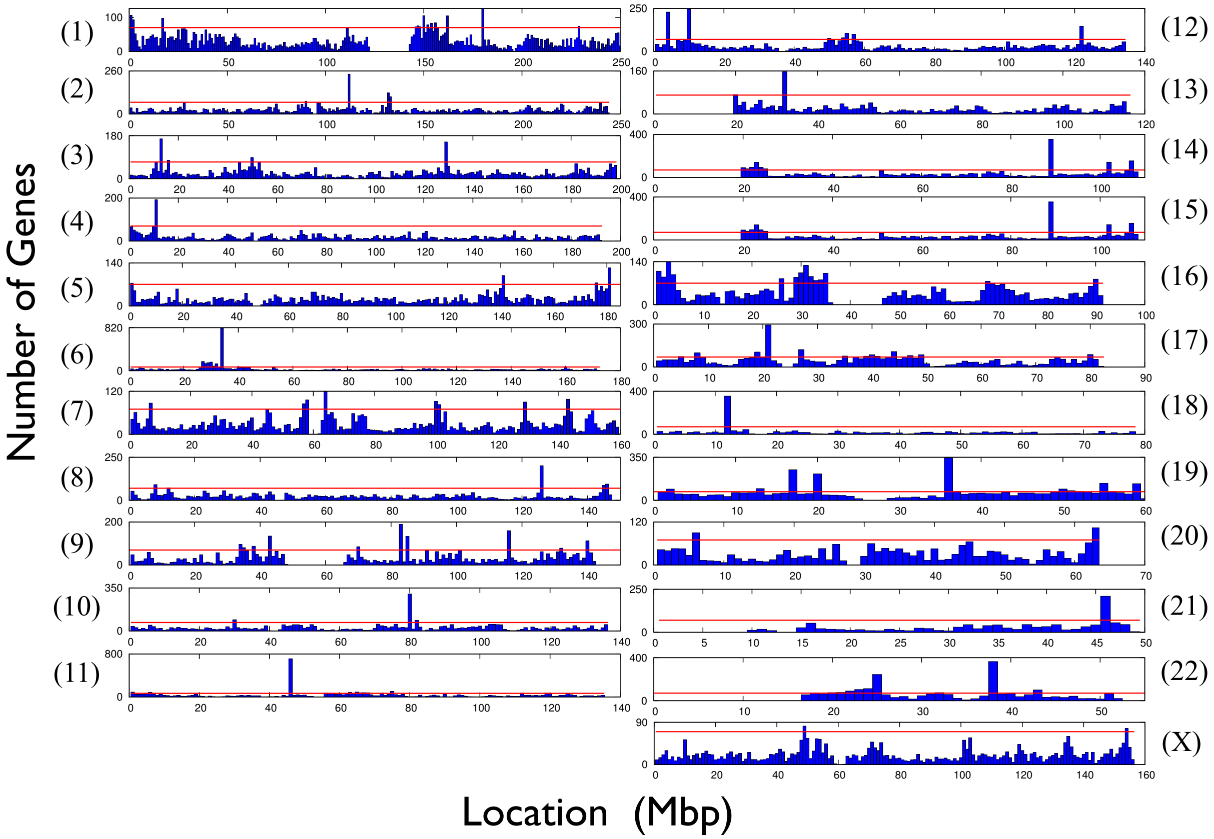

Fig. S 1. Gene density, in 1 MB units, along each of the 22 and X human chromosomes, with data from the Gene Cards database[1, 2]. The horizontal line drawn represents our 5% cutoff for considering a monomer to be active.

defining the ellipsoid in standard form

$$(\frac{x}{a})^2 + (\frac{y}{a})^2 + (\frac{z}{c})^2 = 1. \tag{1}$$

A necessary condition for $\vec{x}$ to be the closest point to $\vec{p}$ is that $\vec{p} - \vec{x}$ is perpendicular to the tangent plane in $\vec{x}$.

It is convenient to define $\bar{r}_i$ through $\bar{r}_i = \sqrt{x_i^2 + y_i^2}$. For points *on* the ellipse obtained in a constant-z cross-section of the ellipsoid, we have $\bar{r}_i = R = a\cos\theta$. This transformation then reduces the prolate or oblate ellipsoid equation to the equation of an ellipse in the $(\bar{r}, z)$ plane of the form

$$(\frac{R}{a})^2 + (\frac{z}{c})^2 = 1. \tag{2}$$

We may now apply standard techniques to minimize

$$\triangle R_i^2 = (\bar{r}_i - a\cos\theta)^2 + (z_i - c\sin\theta)^2$$

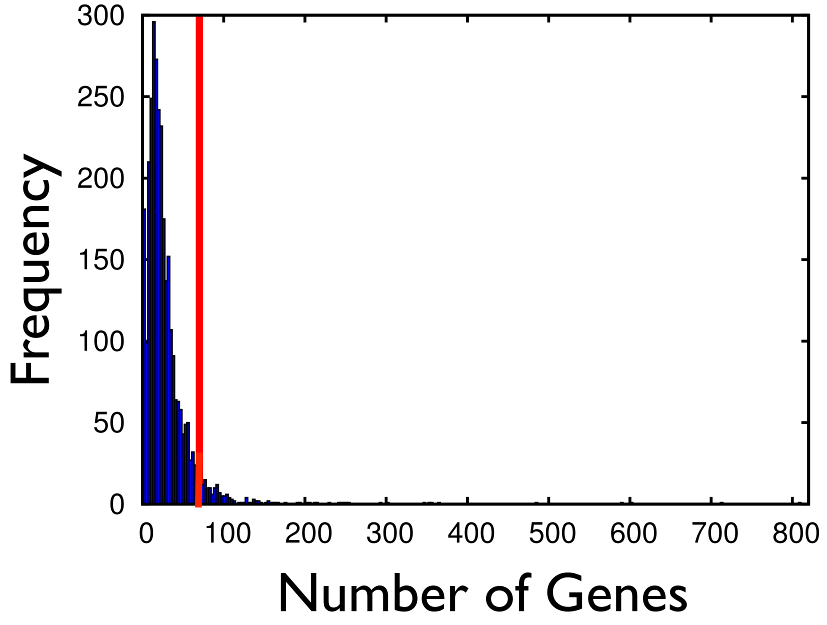

Fig. S 2. Histogram of total gene density per monomer. The cutoff we use to separate the top 5% of genes in terms of gene density is shown as the vertical line.

where we minimize over θ, given $\bar{r}_i, z_i$, requiring that $\frac{d(\triangle R_i{}^2)}{d\theta} = 0$ and $\frac{d^2(\triangle R_i{}^2)}{d\theta^2} > 0$ where

$$\frac{d(\triangle R_i{}^2)}{d\theta} = (a^2 - c^2)\sin\theta\cos\theta - \bar{r}_i\, a\sin\theta + z_i c\cos\theta$$
$$\frac{d^2(\triangle R_i{}^2)}{d\theta^2} = (a^2 - c^2)(\cos^2\theta - \sin^2\theta) - \bar{r}_i\, a\cos\theta - z_i c\sin\theta \tag{3}$$

Solving this system of equations using a bisection method, we obtain a value for θ which minimizes $\triangle R_i^2$. Using this value of θ, the value of $\triangle R_i^2$ is evaluated, enabling a calculation of force components acting on the monomers from the wall, using the formula

$$\begin{aligned} V_{wall} &= (\Delta R_i)^5 \;\; for \Delta R_i \geq 0 \\ &= 0 \qquad\qquad for \;\; \Delta R_i < 0 \end{aligned} \tag{4}$$

III. Active Interactions with the Nuclear Envelope

To model the selective interactions of monomers with the nuclear envelope, we allow the wall to selectively interact with a fraction of the total number of monomers contained within our model nucleus, via a short-range attractive potential. To further simplify, we assign this fraction to the active monomers. We calculate $\triangle R_i^2$, the closest (squared) distance between

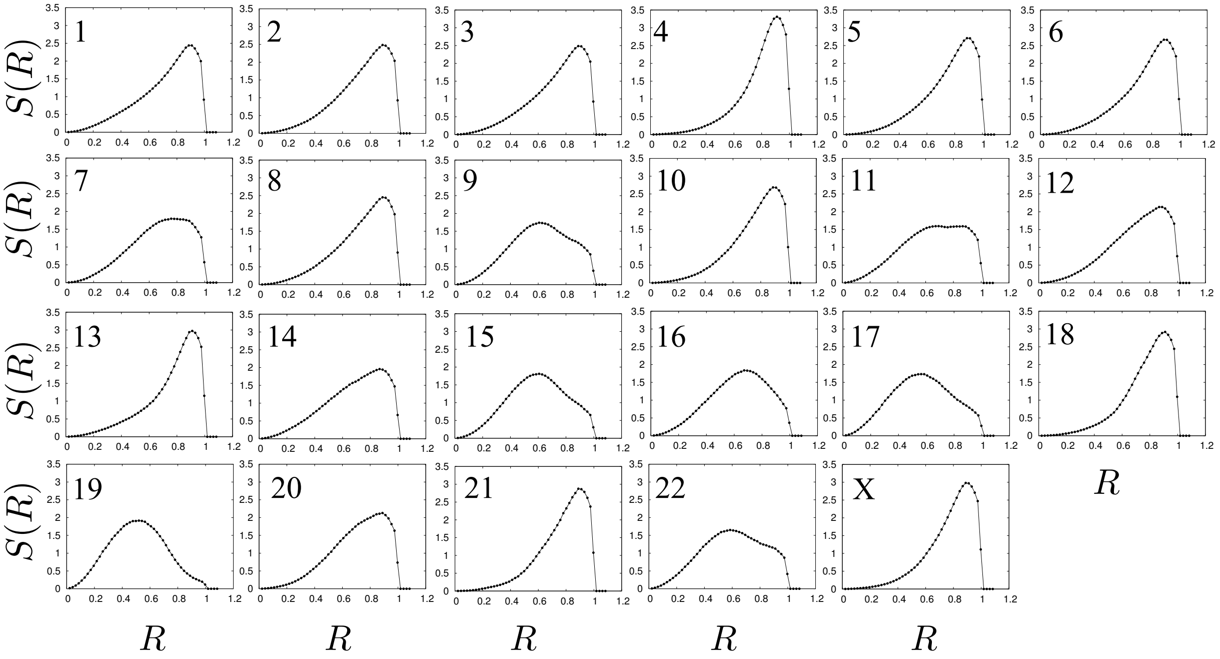

Fig. S 3. $S(R)$ for all chromosomes labeled 1 …22, X, computed for a spherical geometry and for inhomogeneous activity but without random loops, illustrating how such distribution functions vary depending on the size and activity of the chromosome. Of these, we compare distributions for chromosomes 18 and 19 as well as 12 and 20 to experimental data in the main text.

a monomer labelled by i and the nuclear envelope and choose the NE potential for $\Delta R_i \geq 0$ as

$$V_{wall} = (\Delta R_i)^5 \qquad \text{for } \Delta R_i \geq 0 \tag{5}$$

while for $\Delta R_i < 0$, it is

$$\begin{aligned} V_{wall} &= -V_{w0} \exp(-\Delta R_i^2 / 2{\sigma_w}^2) \quad \text{for active monomers} \\ &= 0 \qquad\qquad\qquad \text{for} \quad \text{inactive monomers} \end{aligned}$$

where $V_{w0} = 60\, k_B T_{eq}$ and $\sigma_w^2 = 15.0$. The choice of the depth of the potential well, as well as of its range, is to some extent arbitrary, since this procedure isolates the complexities of chromatin-NE interaction into an extremely simplified form. However, since we expect that such an interaction should be strong enough to be counter the natural tendency of chromosomes to segregate by (inhomogeneous) activity, the depth of this interaction should certainly exceed the upper limit for active temperatures. The relative strength of this effect is then dictated by the Boltzmann factor $\sim \exp(-60 k_B T_{eq}/20 k_B T_{eq}) = \exp(-3) \sim$

0.05, strong enough to produce an appreciable wall effect, yet not so strong as to dominate over the intrinsic tendency towards activity-based segregation. The thickness of the lamins supporting the nuclear envelope is about 40-100 nm, but nuclear lamins also extend into the nucleoplasm and other NE proteins add to this effective thickness; with the assumptions above, the length scale set by the wall interaction in our model is $\sqrt{15} \times 28nm \simeq 108.4nm$

IV. Positioning intermediates obtained as NE interactions are turned on

Given our benchmarking of characteristic time-scales in our problem, we may investigate questions of more dynamical significance, the time it takes for a steady state to respond to a perturbation. To keep our model as simple as possible, we investigate a very specific perturbation, the reorganization of chromosome positions in response to a NE perturbation constituted by an attractive potential interacting only on *active* monomers.The wall interaction is switched on at time $t = 0$, for a set of 10 independently equilibrated series of initial configurations and the evolution of chromosome configurations tracked. $S(R)$ at intermediate times is averaged over the initial configurations.

---

[1] Rebhan, M., Chalifa-Caspi, V., Prilusky, J., and Lancet, D. (1998) GeneCards: a novel functional genomics compendium with automated data mining and query reformulation support.. *Bioinformatics,* **14**(8), 656–664.

[2] Safran, M., Solomon, I., Shmueli, O., Lapidot, M., Shen-Orr, S., Adato, A., Ben-Dor, U., Esterman, N., Rosen, N., Peter, I., Olender, T., Chalifa-Caspi, V., and Lancet, D. (2002) GeneCards 2002: towards a complete, object-oriented, human gene compendium. *Bioinformatics,* **18**(11), 1542–1543.
